# Prior COVID-19 infection may increase risk for developing endothelial dysfunction following hematopoietic cell transplantation

**DOI:** 10.3389/fonc.2022.1000215

**Published:** 2023-01-17

**Authors:** Sydney Ariagno, Dristhi Ragoonanan, Sajad Khazal, Kris M. Mahadeo, Gabriel Salinas Cisneros, Matt S. Zinter, Robyn A. Blacken, Gopi Mohan, Leslie E. Lehmann, Asmaa Ferdjallah, Kristin C. Mara, Mira A. Kohorst

**Affiliations:** ^1^ Pediatric and Adolescent Medicine, Mayo Clinic, Rochester, MN, United States; ^2^ Division of Pediatrics, Stem Cell Transplantation and Cellular Therapy, The University of Texas MD Anderson Cancer Center, Houston, TX, United States; ^3^ Pediatric Hematology and Oncology, University of California San Francisco, San Francisco, CA, United States; ^4^ Pediatric Critical Care Medicine, University of California San Francisco, San Francisco, CA, United States; ^5^ Cancer and Blood Disorders Center, Boston Children’s Hospital, Boston, MA, United States; ^6^ Pediatric Critical Care, Massachusetts General Hospital, Boston, MA, United States; ^7^ Hematology-Oncology, Boston Children’s Hospital, Boston, MA, United States; ^8^ Pediatric Stem Cell Transplant, Dana Farber Cancer Institute/Boston Children’s Hospital, Boston, MA, United States; ^9^ Pediatric Hematology and Oncology, Mayo Clinic, Rochester, MN, United States; ^10^ Department of Quantitative Health Sciences, Mayo Clinic, Rochester, MN, United States

**Keywords:** COVID-19, hematopoietic cell transplant, TA-TMA, VOD/SOS, engraftment syndrome, endothelial dysfunction

## Abstract

Endothelial dysfunction underlies many of the major complications following hematopoietic cell transplantation (HCT), including transplant-associated thrombotic microangiopathy (TA-TMA), veno-occlusive disease/sinusoidal obstruction syndrome (VOD/SOS), and engraftment syndrome (ES). Emerging evidence similarly implicates endothelitis and microangiopathy in severe COVID-19-related multi-system organ dysfunction. Given the overlap in these two illness states, we hypothesize that prior COVID-19 infection may increase risk for HCT-related endotheliopathies. This retrospective, multicenter study included patients aged 0-25 years who underwent autologous or allogeneic HCT for any indication between January 1, 2020 and September 21, 2021, with close attention to those infected with COVID-19 in either the six months prior to transplant or twelve months following transplant. Incidences of TA-TMA, VOD/SOS, and ES were compared among patients with COVID-19 infection pre-HCT and post-HCT, as well as with historical controls who were never infected with SARS-CoV-2. Those who underwent HCT following COVID-19 infection displayed significantly increased rates of TA-TMA compared to those who were never infected. Additionally, our data suggests a similar trend for increased VOD/SOS and ES rates, although this did not reach statistical significance. Therefore, a history of COVID-19 infection prior to undergoing HCT may be a nonmodifiable risk factor for endothelial-related complications following HCT. Further studies are warranted to better clarify this relationship among larger cohorts and in the era of the Omicron SARS-CoV-2 variants.

## 1 Introduction

While scientific knowledge regarding the severe acute respiratory syndrome coronavirus-2 (SARS-CoV-2) has expanded rapidly since the beginning of the coronavirus disease 2019 (COVID-19) pandemic, unanswered questions remain pertaining to the impact of this illness on medically unique populations. Pediatric patients undergoing hematopoietic cell transplantation (HCT) are a medically complex group in which the impact of SARS-CoV-2 infection is not well understood. While pediatric patients in general seem to be at lower risk for severe disease from COVID-19, adults undergoing HCT appear to be at increased risk for COVID-19-related severe disease and mortality (1–3).

Data characterizing the experience of this unique pediatric population is limited and predominately describes patients infected following HCT. Lucchini et al. recount the UK experience, in which the majority of their post-HCT pediatric patients who became infected with SARS-CoV-2 had mild illness, although one of the nine patients did develop severe COVID-19 infection with cytokine-release syndrome (4). A series of more severe cases was described by Barhoom et al., where they detailed four pediatric patients who contracted COVID-19 following HCT, three of whom required endotracheal intubation and ICU level care and two of whom died as a result of the infection (5). The largest study to date includes 62 children infected with SARS-CoV-2 following HCT, with 10% of those patients requiring ICU level care (6). COVID-19-specific mortality among this cohort was 5% (6). In total, most of the available literature highlights COVID-19 infection months following transplant, with little data available regarding patients who acquire SARS-CoV-2 prior to or immediately surrounding HCT. Additionally, no available literature describes the relationship between COVID-19 infection and HCT-specific complications or outcomes.

Mounting evidence suggests that severe disease from SARS-CoV-2 is largely mediated by systemic endothelial injury (7, 8). Analysis of biopsy samples from surviving and postmortem patients with severe COVID-19 infection reveals endothelial destruction in all major organ systems (9–11). Under normal conditions, endothelial cells regulate vascular tone, inflammatory cascades, vessel permeability, and the balance between prothrombic and antithrombic states. In the setting of SARS-CoV-2 infection, endothelial dysfunction likely arises from a combination of two factors – direct viral invasion of endothelial cells and cytokine hyperactivation initiated by pulmonary COVID-19 infection (10–12). Subsequently, diffuse vascular leakiness and interstitial edema, microvascular and macrovascular thromboembolic events, and overwhelming immune activation lead to multisystem organ dysfunction/failure (8, 10, 13).

The effect of COVID-19 infection on endothelial function may persist long after the acute infection has resolved. A study by Chioh et al. demonstrated that there were persistent cellular and biochemical markers of endothelial injury and cytokine hyperactivation among convalescing patients (14). Additionally, in prospective studies examining endothelial function as estimated by brachial artery flow-mediated dilation, multiple groups have demonstrated persistent endothelial dysfunction up to six months following COVID-19 infection (15, 16). These authors hypothesize that this continued endothelial dysfunction may predispose patients with a history of COVID-19 infection to thromboembolic events. Indeed, clinical data is accumulating that some adult patients may experience ongoing risk of vascular complications for weeks after acute SARS-CoV-2 infection (17, 18).

The systemic endothelial dysfunction seen in severe SARS-CoV-2 infection shares a similar pathophysiology with the endotheliopathies seen following HCT (19, 20). This collection of post-HCT complications include transplant associated thrombotic microangiopathy (TA-TMA), veno-occlusive disease/sinusoidal obstruction syndrome (VOD/SOS), engraftment syndrome (ES), graft-versus-host disease (GvHD), idiopathic pneumonia syndrome/diffuse alveolar hemorrhage (IPS/DAH), and thrombosis/coagulopathy. Factors that contribute to endothelial injury in HCT settings include high-intensity conditioning chemotherapy, total body irradiation, certain underlying oncologic diagnoses, degree of alloreactivity, and GvHD prophylactic agents such as calcineurin inhibitors (21). In each of these complications, systemic or organ-specific endothelial damage occurs, leading to increased vascular permeability, decreased regulation of vascular tone, activation of coagulation factors, and excess inflammatory/cytokine signaling (21).

Given these shared mechanisms, as well as the lingering nature of the endothelial dysfunction following COVID-19 infection, we hypothesize that patients with SARS-CoV-2 infection in the time period immediately pre- or post- HCT may be at increased risk of developing HCT-related complications mediated by endothelial dysfunction. Therefore, in this study we assessed the incidence of transplant-related endotheliopathies in patients with COVID-19 infection pre-HCT and post-HCT compared to historical controls without documented SARS-CoV-2.

## 2 Methods

This multicenter study evaluated patients across four geographically diverse academic pediatric centers. All patients aged 0-25 years who underwent autologous or allogeneic HCT for any indication from January 1, 2020 to September 21, 2021 were included. The study group was comprised of all patients with a laboratory confirmed case of COVID-19 in either the six months prior to or twelve months following HCT. Clinical variables of interest were collected *via* retrospective review of the medical record and stored on a de-identified, secure database.

Data was summarized using frequencies and percentages for categorical data and medians with either ranges or interquartile ranges for continuous data. Data was compared between groups (COVID-19 pre-HCT vs COVID-19 post-HCT) using a Fisher’s exact tests for categorical data, and a Wilcox rank sum test for continuous data. All tests were two-sided, and p-values ≤ 0.05 were considered statistically significant. All analyses were performed using R version 4.1.2 (R Core Team, R Foundation for Statistical Computing, Vienna, Austria).

This study was approved by the institutional review boards at each of the participating centers. Patient consent was not required given the de-identified and retrospective nature of the investigation.

## 3 Results

A total of 374 pediatric and young adult patients underwent HCT for any indication during the specified date range across the four sites. Ten patients contracted COVID-19 in the six months prior to HCT, and 13 contracted COVID-19 in the twelve months following HCT. Demographic characteristics of the infected patients are in **Table 1A**. No significant differences in age, sex, race, pre-transplant performance score, or underlying pre-transplant diagnosis were found between the COVID-19 Pre-HCT group and COVID-19 Post-HCT group.

**Table 1A:** Baseline demographic characteristic of HCT patients who contracted COVID-19 pre- and post-transplant.

	All COVID-19 Patients	COVID-19	COVID-19	p-value
	(N=23)	Pre-HCT	Post-HCT	
		(N=10)	(N=13)	
**Age, years** , Median (IQR)	15.0 (5.3, 17.0)	14.6 (5.7, 16.0)	16.0 (5.0, 20.0)	0.71
**Sex (% male)**	61%	70%	54%	0.67
**Race**				0.2
White	48%	60%	38%	
Black or African American	4%	0%	8%	
More than one race	30%	40%	23%	
Unknown	17%	0%	31%	
**Pre-Transplant Performance Score**, Median (range)	90 (50-100)	90 (70-100)	90 (50-100)	0.63
**Underlying Diagnosis**				0.78
AML	30%	30%	31%	
ALL	17%	20%	15%	
MDS/MPN	9%	10%	8%	
Other acute leukemia	4%	0%	8%	
Hodgkin lymphoma	4%	0%	8%	
Solid tumor	9%	20%	0%	
SAA	4%	10%	0%	
Immune disorder	17%	10%	23%	
Other	4%	0%	8%	

Clinical data regarding HCT variables were compiled for patients who contracted COVID-19 (**Table 1B**). Transplant type, number of lifetime transplants, stem cell source, intensity of conditioning regimen, GVHD prophylactic agents, and exposure to serotherapy were similar between the groups. Patients in the COVID-19 Pre-HCT group were significantly more likely to have undergone haploidentical or mismatched transplantation, whereas patients in the COVID-19 Post-HCT group were more likely to have undergone matched transplantation.

**Table 1B:** HCT variables of patients who contracted COVID-19 pre- and post-transplant.

	All COVID-19 Patients (N=23)	COVID-19 Pre-HCT (N=10)	COVID-19 Post-HCT (N=13)	p-value
**Transplant Type**				0.99
Allogeneic	83%	80%	85%	
Autologous	17%	20%	15%	
**Transplant Number**				0.99
First	78%	80%	77%	
Second	13%	10%	15%	
Third	9%	10%	8%	
**Donor Type**				0.02
MRD	17%	0%	31%	
MUD	17%	0%	31%	
Haploidentical	26%	50%	8%	
MMUD	17%	30%	8%	
UCB	4%	0%	8%	
Autologous	17%	20%	15%	
**Stem Cell Source**				0.81
Bone marrow	48%	40%	54%	
PBSC	48%	60%	38%	
UCB	4%	0%	8%	
**Conditioning Regimen**				0.8
Myeloablative	70%	80%	62%	
RIC	26%	20%	31%	
Non-Myeloablative	4%	0%	8%	
GVHD Prophylaxis (may have received more than one)				
*Ex-vivo* T cell depletion	13%	20%	8%	0.56
CD34 selection	0%	0%	0%	--
PTCY + other	26%	30%	23%	0.99
PTCY alone	0%	0%	0%	--
TAC/CSA + MTX +/- other	26%	30%	23%	0.99
TAC/CSA + MMF +/- other	35%	60%	31%	0.22
TAC/CSA + other	9%	0%	15%	0.49
TAC/CSA alone	0%	0%	0%	--
Other	4%	0%	8%	0.99
None	13%	20%	8%	0.56
**Serotherapy**				0.57
ATG	30%	30%	31%	
Alemtuzumab	9%	0%	15%	
None	61%	70%	54%	

COVID-19 infection characteristics were compiled (**Table 2**). Median date of COVID-19 diagnosis for the Pre-HCT group was day -107.5 (range: -73 to -205); the Post-HCT median infection date was day +206 (range: +54 to +345). Symptoms of COVID-19 infection were largely similar between groups, except for gastrointestinal symptoms, which were more likely to be reported in the Pre-HCT group. Rates of asymptomatic infection were similar between groups (20% vs. 31%, p = 0.66), as was need for intensive care (10% vs. 15%, p = 0.99).

**Table 2:** SARS-Cov-2 infection details among HCT patients who contracted COVID-19 pre- and post-transplant.

	All COVID-19 Patients	COVID-19	COVID-19	p-value
	(N=23)	Pre-HCT	Post-HCT	
		(N=10)	(N=13)	
**Date of Diagnosis Relative to Day 0**, Median (range)	120 (-205 to 345)	-107.5 (-73 to -205)	206 (54 to 345)	
COVID-19 Symptoms (may have had ≥ 1 symptom))				
Pyrexia	43%	60%	31%	0.22
Respiratory	65%	60%	69%	0.69
Cardiovascular	4%	0%	8%	0.99
Gastrointestinal	17%	40%	0%	0.024
Headaches	9%	20%	0%	0.18
Other neurological	9%	0%	15%	0.49
Coagulopathy	4%	10%	0%	0.43
Loss of taste and/or smell	0%	0%	0%	--
Asymptomatic	26%	20%	31%	0.66
Other	0%	0%	0%	--
**ICU Level Care Required**	13%	10%	15%	0.99
COVID-19 Directed Therapies (may have received more than one)				
Convalescent plasma	13%	30%	0%	0.068
Monoclonal antibody	9%	10%	8%	0.99
Remdesivir	30%	50%	15%	0.17
Steroid	26%	40%	15%	0.34
Tocilizumab	0%	0%	0%	--
Azithromycin	4%	10%	0%	0.43
Other antibiotic	17%	20%	15%	0.99
Other antiviral	0%	0%	0%	--
Other	13%	20%	8%	0.56
None	61%	50%	69%	0.42

Incidence of TA-TMA, VOD/SOS, and ES were compared between patients infected with COVID-19 Pre- and Post-HCT, as well as to the larger group of historical controls who were never infected with COVID-19 (**Table 3**). Patients in the Pre-HCT group were significantly more likely to experience TA-TMA as compared with patients who were never infected with COVID-19 (40% vs. 4.6%, p = 0.003). Several other non-statistically significant trends were noted as well. Incidence of TA-TMA may be higher among the Pre-HCT patients as compared to Post-HCT patients (40% vs. 8%, p = 0.20). VOD/SOS may occur more frequently among the Pre-HCT patients, as compared to the Post-HCT patients (30% vs. 8%, p = 0.42) and the non-COVID patients (30% vs. 14.8%, p = 0.42). ES displayed similar non-statistically significant trends. Incidence among the Pre-HCT patients may be increased as compared to the Post-HCT patients (20% vs. 0%, p = 0.35) and the non-COVID patients (20% vs. 8.8%, p = 0.35). Rates of VOD/SOS and ES were lowest in the Post-HCT patients (8%, 0%), which may be a reflection of small sample size.

**Table 3:** Incidences of TA-TMA, VOD/SOS, and ES among HCT patients who contracted COVID-19 pre-transplant, post-transplant, or were never infected.

	COVID-19	COVID-19	Non-COVID	Pre vs Post	Pre vs Non	Post vs Non
	Pre-HCT	Post-HCT	HCT Patients (n=351)	p-value	p-value	p-value
	(n=10)	(n=13)				
**TA-TMA**	40% (n=4)	8% (n=1)	4.56% (n=16)	0.2	0.003	0.47
**VOD/SOS**	30% (n=3)	8% (n=1)	14.81% (n=52)	0.42	0.42	0.7
**ES**	20% (n=2)	0% (n=0)	8.83% (n=31)	0.35	0.35	0.61

Individual case details for the 10 patients in the COVID-19 Pre-HCT group are outlined in **Table 4**. Two patients died (20%); one death was attributed to multi-system organ failure in the setting of multiple transplant-related endotheliopathies. The other death was due to multi-system organ failure from both COVID-19 infection and multiple transplant-related endotheliopathies. Among the patients who were in the COVID-19 Post-HCT group, two patients died (15.4%). Neither death was attributed to COVID-19 infection or transplant-related endotheliopathy.

**Table 4:** Case details of all HCT patients who were infected with COVID-19 pre-transplant.

Patient #	Age (years)	M/F	Underlying Diagnosis	Transplant Type	Conditioning	GVHD Prophylaxis	COVID-19 Date of Diagnosis	COVID-19 Symptoms	COVID-19 Directed Therapies	Need for ICU level care for COVID-19	Coagulopathy	TA-TMA	VOD/SOS	Engraftment syndrome	Acute GVHD (Grade II-IV)	Chronic GVHD	Vital Status	Cause of death
1	5	M	Severe aplastic anemia	Allogeneic	RIC	T-cell depletion, MMF	-205	Pyrexia, GI, headaches	None	No	No	Yes	No	No	No	No	Alive	Not applicable
2	3	M	Neuroblastoma	Autologous	Myeloablative	None	-118	Asymptomatic	None	No	No	Yes	Yes	Yes	No	No	Deceased	Multi-system organ failure secondary to DAH, TA-TMA, VOD/SOS
3	14	M	Ewing sarcoma	Autologous	Myeloablative	None	-79	Asymptomatic	None	No	No	No	No	No	No	No	Alive	Not applicable
4	24	F	AML	Allogeneic	Myeloablative	T-cell depletion	-80	Respiratory	Remdesivir, steroids	No	No	No	No	No	No	No	Alive	Not applicable
5	16	M	ALL	Allogeneic	Myeloablative	PTCY, Tacrolimus, MMF	-134	Pyrexia, respiratory	Convalescent plasma, remdesivir	No	No	No	Yes	No	No	No	Alive	Not applicable
6	17	M	ALL	Allogeneic	Myeloablative	Tacrolimus, mini-MTX	-153	Coagulopathy	Convalescent plasma, monoclonal antibody, remdesivir, steroids, azithromycin	No	No	No	No	No	Yes	No	Alive	Not applicable
7	6	F	Immune disorder	Allogeneic	RIC	PTCY, Tacrolimus, MMF	-163	Pyrexia, respiratory, GI	None	No	No	Yes	Yes	Yes	No	Yes	Alive	Not applicable
8	16	M	AML	Allogeneic	Myeloablative	PTCY, Tacrolimus, MMF	-96	Pyrexia, respiratory	Remdesivir, steroids	No	No	No	No	No	No	No	Alive	Not applicable
9	15	F	Myelodysplastic syndrome	Allogeneic	Myeloablative	Yes	-97	Pyrexia, respiratory, GI, headaches	Convalescent plasma, remdesivir, steroids, Anakinra	Yes	Yes	Yes	No	No	Yes	No	Deceased	Multi-system organ failure secondary to either protracted initial or secondary COVID-19 infection
10	2	M	AML	Allogeneic	Myeloablative	Tacrolimus, mini-MTX, Abatacept	-73	Pyrexia, respiratory, GI	None	No	No	No	No	No	No	No	Alive	Not applicable

## 4 Discussion

In this study, we sought to understand the impact of COVID-19 infection on pediatric HCT outcomes, particularly transplant-associated endotheliopathies. Among this small group of pediatric and young adult patients, those who underwent HCT following COVID-19 infection displayed significantly increased rates of TA-TMA compared to those who were infected after transplant or historical controls who were never infected. Additionally, our data suggests a similar trend for increased VOD/SOS and ES rates, although this did not reach statistical significance. We do acknowledge that rates of VOD/SOS and ES were lowest in our cohort of patients who contracted COVID-19 following HCT, however we suspect this is due to small sample size and low statistical power rather than a true protective effect. Finally, for both patients from the COVID-19 Pre-HCT cohort who died, multiple identified endotheliopathies contributed to mortality.

To our knowledge, no other studies have reported on HCT-associated outcomes in pediatric patients with a history of prior COVID-19 infection. However, there are a few cases in the literature that support the pathophysiologic link between transplant endothelitis and COVID-19 endothelial dysfunction. Among the patients described by Lucchini et al., one patient who had mild illness from COVID-19 infection on day +138 went on to develop TA-TMA, in the absence of other commonly accepted TA-TMA risk factors (4). The authors suggest that COVID-19 infection was the suspected trigger for endothelial derangement in this patient. In the same cohort, the only patient who experienced severe illness and cytokine release syndrome from COVID-19 infection had undergone HCT for sickle cell anemia, specifically due to cerebrovascular disease which may suggest pre-existing endothelial dysfunction (4). Additionally, one patient reported by Barhoom et al. who developed COVID-19 infection on day +27 of transplant course developed TA-TMA within days of the infection and ultimately died as a result of this complication (5).

Evidence to support the increased risk of TA-TMA among transplant recipients who experience COVID-19 infection reaches beyond the realm of HCT. Two cases of TMA concurrent with COVID-19 infection in renal transplant recipients have been reported. The first describes an adult patient who was nine years post-allograft receipt and developed TMA 11 days following COVID-19 diagnosis; other known causes of TMA were ruled out. His course was also characterized by multisystem organ dysfunction and myocardial infarction, suggestive of global endothelial dysfunction (22). The second patient contracted COVID-19 three months following renal transplantation; acute kidney injury, hemolytic anemia, and coagulopathy were the presenting features of her COVID-19 diagnosis. Renal biopsy confirmed the diagnosis of TMA, with SARS-CoV-2 infection named as the suspected cause (23).

The shared pathogenic mechanism between severe COVID-19 infection and post-HCT endotheliopathies may be rooted in excess complement activation. SARS-CoV-2 infections have been shown to cause aberrant complement activation *via* the classical, lectin, and alternative complement pathways (24, 25). Genetic differences in the individual components of the complement system have been proposed as a contributing factor to varying illness severity amongst patients with COVID-19. In particular, several polymorphisms of the MBL allele, which codes for the MBL protein of the lectin pathway, are known to cause excess complement activation and are associated with poor clinical outcomes in setting of severe infections (25). Similarly, excess complement activation is a contributing cause to post-HCT endothelial dysfunction, particularly TA-TMA. A number of pathogenic variants in a variety of complement factors have been identified in association with increased risk for TA-TMA (25–27). Further investigation is recommended to evaluate for unifying genetic predisposition for both conditions. Greater understanding of the genetic underpinnings for excess complement activation may eventually impact clinical guidance for increased screening, personalized transplant protocols, and therapeutic strategies for affected individuals.

Additionally, excess and prolonged cytokine release is likely a shared pathophysiologic mechanism that unifies severe COVID-19 illness and HCT-associated endotheliopathies. IL-6, IL-1β, and TNF-α have been named as key cytokines that become overexpressed in the setting of severe COVID-19 infection (28). Particularly for the pediatric population, these are key cytokines involved in the development of the multisystem inflammatory syndrome in children (MIS-C) that arises after COVID-19 infection. Specific therapeutic targets such as tocilizumab and anakinra are being investigated for their use in severe COVID-19 illness, due to their effects of modulating excess cytokine signaling. Likewise, IL-6 and TNF-α, in addition to a number of other cytokines, are excessively active in patients who have developed HCT-associated endothelial complications (21, 29). The use of targeted cytokine antagonists in preventing or treating HCT-associated endotheliopathies has been suggested in the literature, however to our knowledge has not been explored in a large-scale trial. Further investigation into cytokine signaling among patients undergoing HCT in close temporal relation to SARS-CoV-2 infection is warranted to better understand this relationship.

Two clinical applications of interest arise from these findings. First, our reported findings may suggest that prior COVID-19 infection could be a nonmodifiable risk factor for transplant-associated endotheliopathy. As such, institutions may consider implementing additional screening for affected patients throughout their transplant process. For example, we endorse the TA-TMA screening guidelines recommended by Dandoy et al., which includes twice weekly lactate dehydrogenase measurement and weekly urinalysis with urine protein creatinine ratio for the first 30 days post-HCT. For patients undergoing HCT for non-malignant and non-urgent indications who contract COVID-19 prior to HCT, consideration may be given to delaying HCT. The appropriate interval for delaying HCT is not yet defined, and further investigation is required prior to formalizing this recommendation (30). Second, recognition of the overlapping risks between HCT-related endotheliopathies and COVID-19 endothelial dysfunction raises the question of shared therapeutics between the two diseases. Prior researchers have called for studies investigating the use of agents for VOD/SOS and TA-TMA for severe COVID-19. Both defibrotide and eculizumab have demonstrated improved short- and long-term outcomes without significant adverse effects among patients with severe SARS-Cov-2 infection (31, 32).

Currently, the impact of SARS-CoV-2 infection on success of HCT engraftment and ultimate outcome is unknown. Key endothelial cell populations are thought to be involved in homing, engraftment, and restoration of bone marrow functioning following HCT (33, 34). Damage to crucial endothelial cells, such as by recent COVID-19 illness, may conceivably impair success of HCT engraftment. Further investigation into long term transplant outcomes amongst patients with recent SARS-CoV-2 infection who undergo HCT is imperative to understand this relationship.

The following limitations must be considered while interpreting the results of this study. First, although this study was conducted across four pediatric HCT centers, the frequency of patients infected with COVID-19 in the peri-HCT period was low. The sample size of our population of interest is small, which limits study power and ability to perform complete statistical analyses, including multivariate analysis. We strongly encourage ongoing investigation regarding this topic, as more patients will be available for study as the COVID-19 pandemic progresses. Second, he patients included in this research contracted COVID-19 prior to the emergence of the Omicron variants. The Omicron variant infected significantly more pediatric patients nationwide, however severe clinical outcomes were fewer as compared to pediatric patients infected with the Delta variant (35). Follow up studies are recommended to capture the impact of the Omicron strain, as well as future variants that may arise. Third, data regarding patient serologic status and evidence of prior COVID-19 infection was not available for our patient cohort, as these patients were studied prior to the widespread availability of serologic COVID-19 testing. Therefore, we do not have insight into whether these patients experienced a primary or subsequent COVID-19 infection and therefore cannot comment on the impact of subsequent infections on risk of developing endothelial complications. Fourth, we acknowledge a major potential confounder in our underlying HCT variables; patients who contracted COVID-19 prior to HCT were more likely to receive haploidentical grafts, as compared to patients who contracted COVID-19 following HCT. Increased degree of alloreactivity, as well as differences in conditioning and immunosuppression that coincide with haploidentical transplantation, are known risk factors for endothelial complications following HCT (36). Similar studies with larger cohorts are warranted to more effectively control for confounders.

## Data availability statement

The raw data supporting the conclusions of this article will be made available by the authors, without undue reservation.

## Ethics statement

The studies involving human participants were reviewed and approved by Mayo Clinic IRB, UCSF IRB, MD Anderson Cancer Center IRB, Dana Farber/Boston Children’s IRB. Written informed consent from the participants’ legal guardian/next of kin was not required to participate in this study in accordance with the national legislation and the institutional requirements.

## Author contributions

SA, MK, KMM, MZ, LL designed the research. SA, DR, SK, GC, RB, GM, AF, MK collected the data. SA, KM, MK analyzed the data. SA, MK wrote the paper. All authors provided edits for the paper. SA and MK directly accessed and verified the underlying data reported in the manuscript. All authors should confirm that they had full access to all the data in the study and accept responsibility to submit for publication. All authors contributed to the article and approved the submitted version.
